# Impact of the COVID-19 Pandemic and Other Factors on Medical Specialty Choice Among Medical Students Near the United States-Mexico Border

**DOI:** 10.7759/cureus.113886

**Published:** 2026-08-03

**Authors:** Ameen Khan, Rebecca Campos, Narges Khanjani

**Affiliations:** 1 Department of Medical Education, Paul Foster School of Medicine, Texas Tech Health El Paso, El Paso, USA

**Keywords:** covid-19, medical field, medical students, specialty, us-mexico border

## Abstract

Background: This study investigated the factors that contribute to medical students' decisions to pursue different residency specialties near the US-Mexico border and the effects of COVID-19 on their specialty selection.

Methods: The study population consisted of current medical students (classes of 2025-2028) attending the Paul Foster School of Medicine. An electronic Qualtrics survey was used to collect anonymous data, including demographics, the impact of COVID-19, and future career interests.

Results: The majority of participants reported that the COVID-19 pandemic had affected them personally and professionally. However, most felt that the pandemic had not affected their desire or attitude toward pursuing a career in medicine or their medical school experience. Students who were parents and those more concerned about workload/work flexibility were more likely to choose Pediatrics. Students who thought the pandemic had affected their medical school experience were less likely to choose Internal Medicine. Students who thought the pandemic had changed the factors they prioritize when choosing a medical specialty and those less concerned about a flexible lifestyle were more likely to choose Surgery. Students with more years of professional work experience were less likely to choose Emergency Medicine, and students more concerned about personal or professional support were more likely to choose Psychiatry.

Conclusion: The COVID-19 pandemic did not significantly affect medical students' residency specialty choices near the United States-Mexico border. Further research involving a broader population is needed to better understand the effects of the pandemic and whether factors are weighed differently across regions of the United States.

## Introduction

The United States (US) is facing a shortage of primary care (PC) physicians, and the factors that attract medical students to different fields can have many implications for the overall healthcare system and its workforce [1]. Additionally, it has been noted that the misalignment between societal needs and student specialty preferences will continue to negatively impact healthcare both currently and in the future [2]. Furthermore, physician burnout and dissatisfaction, which could presumably occur from choosing a particular specialty without being fully informed, have been shown to result in increased medical errors, a declining workforce, and even compromised patient safety [3]. These issues are particularly important near the US-Mexico border, where there is already a physician shortage and a large underserved population. Factors that influence medical specialty choice have been a topic of study in the past; however, the effects of the COVID-19 pandemic on medical students' specialty choices have not been extensively investigated. Some studies show that the COVID-19 pandemic disrupted medical education and affected medical students’ career choices, and these effects may vary based on demographics, such as gender [4]. Now that the COVID-19 pandemic has subsided, this study further investigates the factors that are important to medical students near the US-Mexico border when choosing a medical specialty and the effects that the COVID-19 pandemic has had on those factors. While studies have explored the COVID-19 pandemic’s impact on medical education, few have examined how it shaped specialty decision-making in underserved border regions.

The first aim of this study was to assess the factors that influence medical students’ choice of medical specialty near the US-Mexico border. There is a need to understand the factors that medical students consider when choosing a medical specialty, particularly in underserved regions such as the US-Mexico border. This understanding can help inform targeted career education and advising, as well as strategies to improve physician retention in the region.

The second aim was to evaluate whether the COVID-19 pandemic impacted these factors in medical students’ decision-making processes. Given the disruptions caused by the COVID-19 pandemic, we sought to determine whether the factors that influence specialty selection were affected by personal, professional, or educational changes.

The abstract of this article was presented at the International Association of Medical Science Educators (IAMSE) Annual Conference, held in Calgary, Alberta, Canada, June 14-17, 2025.

## Materials and methods

In this cross-sectional study, the study population consisted of current medical students (classes of 2025-2028) attending Texas Tech University Health Sciences Center (TTUHSC) El Paso Paul L. Foster School of Medicine. There were no specific inclusion or exclusion criteria. All students in the 2025-2028 cohorts at Paul L. Foster School of Medicine were invited to participate. The number of students in each cohort was as follows: 117 in the Class of 2025, 124 in the Class of 2026, 124 in the Class of 2027, and 124 in the Class of 2028. Official Texas Tech University email distribution lists were used to ensure that currently enrolled students had access to the survey. The primary investigator obtained access to the email distribution lists through the Department of Medical Education.

An electronic survey was designed using the Qualtrics platform and distributed to all medical students. Anonymous, nonidentifiable data were collected, including demographics (gender, age, race, marital status, parental status, year of study, highest level of education, and prior professional work experience), the impact of COVID-19, and future career interests. The questionnaire included items adapted from previous studies, as well as new questions developed by the investigators. The questionnaire was piloted on a small group of students. Survey question types included single-select multiple-choice questions, multiple-select multiple-choice questions, rating-scale questions using a 0-7 scale, and an open-ended question asking participants to list their top three fields of consideration.

Questions of special interest regarding the effects of COVID-19 assessed the impact of the pandemic on attitudes toward pursuing hospital or critical care medicine, the desire to pursue a career in medicine, personal or professional impacts, and whether the pandemic changed the factors prioritized when choosing a medical specialty. Respondents were also asked to rate, using a scale, their level of concern regarding workload/work flexibility, support (personal or professional), control and autonomy, maintaining healthy relationships, documentation responsibilities, and liability/malpractice.

A one-time electronic survey was distributed by email. The survey was closed after the predetermined time frame. Some responses were manually transformed to facilitate statistical analysis, which included descriptive statistics, the Pearson chi-square test, Fisher's exact test, and the independent-samples Mann-Whitney U test. Calculations were performed using IBM SPSS Statistics version 29 (IBM Corp., Armonk, NY, USA) or the online calculator available through the Social Science Statistics website (https://www.socscistatistics.com/).

Missing data were excluded from the statistical analyses. Adjustment for multiple statistical comparisons was not performed because of the small sample size.

Responses to the open-ended question asking participants to list their top three fields of consideration were manually grouped by medical specialty for further analysis. The specialties most frequently listed among the top three choices were analyzed further. Cramér's V was calculated as the effect size for chi-square tests. When Fisher's exact test was used, an effect size was not calculated because there is no widely accepted method for calculating an effect size for this test. For the Mann-Whitney U tests, the effect size was calculated as the absolute value of r = Z/√N, where Z is the standardized test statistic, and N is the total number of participants.

## Results

The demographic characteristics of the participants are shown in Table 1. There were 64 survey responses, with approximately 34% (22) coming from MS1 students (year one medical students), and the remaining responses were relatively evenly distributed among MS2-MS4 students (year two to year four medical students). Almost 60% (38) of respondents were female. Most participants reported more than one year of work experience, which primarily consisted of direct patient care. The majority of respondents (37.5% (24)) reported a minimum acceptable annual income between $200,000 and $299,999, with more than 80% (52) reporting a minimum acceptable annual income of $150,000-$300,000+. The most important reported concern was the ability to maintain healthy relationships with people outside the work environment. Finally, the top fields of interest were Internal Medicine (IM; including subspecialties), Emergency Medicine (EM), and Psychiatry, Family Medicine (FM), and Obstetrics & Gynecology (OBGYN) (tied) (Table 1).

**Table 1:** Demographic characteristics of the medical students who participated in this study MS, medical students

Variable	Number (%)
Gender	
Male	25 (39.1%)
Female	38 (59.4%)
Missing	1 (1.6%)
Age	
18-23	9 (14.1%)
24-26	27 (42.2%)
27+	28 (43.8%)
Race	
Asian/Asian American	16 (25.0%)
Black/African American	4 (6.3%)
White	23 (35.9%)
Hispanic/Latin American	18 (28.1%)
Prefer not to respond/other	3 (4.7%)
Marital status	
Single	43 (67.2%)
Married	18 (28.1%)
Other	2 (3.1%)
Missing	1 (1.6%)
Parental status	
Children	5 (7.8%)
No children	59 (92.2%)
Year of study	
MS 1	22 (34.4%)
MS 2	13 (20.3%)
MS 3	15 (23.4%)
MS 4	14 (21.9%)
Highest level of education obtained thus far	
Bachelor's degree	48 (75%)
Master's degree	16 (25%)
Any prior professional work experience?	
Yes	45 (70.3%)
No	19 (29.7%)
How many years of professional work experience?	
<1 year	7 (10.9%)
>1 year	38 (59.4%)
Missing	19 (29.7%)
The student’s most significant work experience (student could choose more than one)	
Direct patient care	26 (40.6%)
Administrative	11 (17.2%)
Non-healthcare or non-medical work experience	16 (25.0%)
Research	11 (17.2%)
Missing	19 (29.7%)
The COVID-19 pandemic has affected the student’s experience while in medical school (remote learning, additional calls, etc.)	
Yes, positive impact	12 (18.8%)
Yes, negative impact	13 (20.3%)
No impact	38 (59.4%)
Missing	1 (1.6%)
The COVID-19 pandemic has affected the student’s attitude to pursue a career in hospital medicine and/or critical care medicine	
Yes, positive impact	12 (18.8%)
Yes, negative impact	12 (18.8%)
No impact	38 (59.4%)
Missing	2 (3.1%)
The COVID-19 pandemic has affected the student’s desire to pursue a career in medicine	
Yes, positive impact	18 (28.1%)
Yes, negative impact	4 (6.3%)
No impact	41 (64.1%)
Missing	1 (1.6%)
The COVID-19 pandemic impacted the student personally	
Yes	41 (64.1%)
No	22 (34.4%)
Missing	1 (1.6%)
The COVID-19 pandemic impacted the student professionally?	
Yes	42 (65.6%)
No	21 (32.8%)
Missing	1 (1.6%)
What minimum income level is acceptable to the student as a practicing physician?	
<$99,999	1 (1.6%)
$100,00-$149,999	7 (10.9%)
$150,000-$199,999	14 (21.9%)
$200,000-$299,999	24 (37.5%)
$300,000+	14 (21.9%)
No preference	3 (4.7%)
Missing	1 (1.6%)
Would the student like to be employed or be part of a private/group practice	
Employed	15 (23.4%)
Private/group practice	19 (29.7%)
Unsure	28 (43.8%)
Missing	2 (3.1%)
Does the student have a desire to practice in a specific setting	
Urban	18 (28.1%)
Rural	5 (7.8%)
Suburban	14 (21.9%)
Unsure	26 (40.6%)
Missing	1 (1.6%)
Which type of practice interests the student (participant could choose more than one)	
Academic (research, clinical, teaching)	38 (59.4%)
Private practice	47 (73.4%)
Group practice	48 (75.0%)
Concierge medicine	10 (15.6%)
Hospital-based	46 (71.9%)
Locum tenens	11 (17.2%)
Consulting	17 (26.6%)
Administrative (director, leadership roles, etc.)	13 (20.3%)
Other non-clinical (public health, etc.)	12 (18.8%)
Missing	1 (1.6%)
Expected total educational debt (undergraduate + graduate school/medical school)	
No debt ($0)	10 (15.6%)
$1-$99,999	17 (26.6%)
$100,000-$149,999	11 (17.2%)
$150,000-$199,999	11 (17.2%)
$200,000-$249,999	8 (12.5%)
$250,000-$299,999	4 (6.3%)
$300,000-$349,999	2 (2.1%)
Missing	1 (1.6%)
The pandemic has changed the factors the student prioritizes when choosing a field of medicine	
Yes	21 (32.8%)
No	42 (65.6%)
Missing	1 (1.6%)
The factors that the student would want to learn more about during their education: (participant could choose more than one)	
Debt repayment programs	37 (15.23%)
Employment vs. private/group practice	51 (20.99%)
Practice setting (rural, urban, etc.)	34 (13.99%)
Types of practices (academic, group, concierge, locum tenens, etc.)	47 (19.34%)
Incorporating community outreach	29 (11.93%)
Liability and malpractice	43 (17.70%)
Other	2 (0.82%)
What areas of medicine interest the student (student could choose up to 3)	
Internal Medicine	14 (10.00%)
Emergency Medicine	11 (7.86%)
Psychiatry	10 (7.14%)
Family Medicine	10 (7.14%)
Obstetrics & Gynecology	10 (7.14%)
Pediatrics	9 (6.43%)
General Surgery	8 (5.71%)
Cardiology	7 (5.00%)
Pathology	6 (4.29%)
Radiology	6 (4.29%)
Orthopedics	5 (3.57%)
Gastroenterology	4 (2.86%)
Hematology/Oncology	3 (2.14%)
Neurology	3 (2.41%)
Anesthesiology	3 (2.14%)
Administrative	3 (2.14%)
Endocrinology	3 (2.14%)
Critical Care	3 (2.14%)
Public Health	2 (1.43%)
Ear, Nose, and Throat	2 (1.43%)
Internal Medicine + Psychiatry dual	1 (0.71%)
Pulmonology	1 (0.71%)
Interventional Cardiology	1 (0.71%)
Gynecology Oncology	1 (0.71%)
Radiology Oncology	1 (0.71%)
Pediatric Oncology	1 (0.71%)
Rural Health	1 (0.71%)
Preventive Lifestyle Medicine	1 (0.71%)
Dermatology	1 (0.71%)
Neonatal Intensive Care Unit	1 (0.71%)
Sports Medicine	1 (0.71%)
Ophthalmology	1 (0.71%)
Hospitalist	1 (0.71%)
Research	1 (0.71%)
Allergy & Immunology	1 (0.71%)
Palliative Care	1 (0.71%)
Interventional Medicine	1 (0.71%)
Missing	11 (7.86%)
Total	64

Regarding the effect of COVID-19, about 60% (38) of respondents stated that the COVID-19 pandemic had no impact on their medical school experience, almost 60% (38) reported no impact on their attitude to pursue a career in hospital medicine or critical care medicine, and 64% (41) reported no impact on their desire to pursue a career in medicine. Among those who stated that the pandemic affected their desire to pursue a career in medicine, the majority (28% of all respondents [18]) reported a positive impact (Table 1).

A summary of student rankings of factors important in decision-making and their concerns is presented in Table 2.

**Table 2:** Summary of student rankings of factors important in decision-making and their concerns

Variable	Median (IQR)
Rank of the level of importance the factor weighs in students' decision (on a scale from 0 to 7)	
It is important for me to choose a field that will provide prestige/respect within the medical community	4 (3, 5)
It is important for me to choose a field in medicine that will provide a flexible lifestyle	6 (5, 7)
Rank of the level of concern the student feels about the following factors (on a scale from 0 to 7)	
Workload/work flexibility	5 (4.75, 6)
Support (personal or professional)	5 (3, 6)
Control and autonomy	5 (4, 6)
Maintaining healthy relationships with family, friends, loved ones outside of work	7 (6, 7)
Documentation and administrative responsibilities	4 (2, 4.75)
Liabilities and malpractice	4 (2, 5)

Responses were also analyzed within the context of specific specialty fields. Among students pursuing Pediatrics, those who were parents were more likely to choose Pediatrics (p<0.001). Among students who did not choose Pediatrics, 7% (3) were parents, whereas among those who did choose Pediatrics, 87% (7) were parents (Table 3). Additionally, concern about workload and work flexibility was greater among students interested in Pediatrics than among other groups (p=0.039) (Table 4).

**Table 3:** The factors associated with choosing Pediatrics (including Pediatrics, Pediatric Oncology, and NICU) as a specialty among medical students at PLFSOM, excluding participants with missing data NICU, Neonatal Intensive Care Unit; MS, medical students; PLFSOM, Paul L. Foster School of Medicine *Fisher's exact test was done **“Unsure” row was not included in this analysis

Variable	Did not choose Pediatrics	Chose Pediatrics	Chi-square	Degrees of freedom	p-value	Cramer’s V effect size
Gender						
Male	17	4	*	1	1.00*	-
Female	25	6				
Age						
18-27	24	5	0.1107	1	0.739	0.056
27+	19	5				
Ethnicity						
Asian/Asian American+Black/African American	13	4	*	3	0.717*	-
White+Hispanic/Latin American	27	6				
Marital status						
Single	27	5	*	1	1.00*	-
Married	15	3				
Parental status						
Children	3	7	*	1	<0.001*	-
No children	39	1				
Year of study						
MS1/MS2	23	5	*	1	1.00*	-
MS3/MS4	19	3				
Highest level of education obtained thus far						
Bachelor's degree	31	7	*	1	0.661*	-
Master's degree	11	1				
Do you have prior professional work experience?						
Yes	15	3	*	1	1.00*	-
No	27	5				
How many years of professional work experience?						
<1 year	4	1	*	1	1.00*	-
>1 year	24	6				
How would you categorize your most significant work experience? (choose all that apply)						
Direct patient care						
Yes	13	4	*	1	0.659*	-
No	13	2				
Administrative						
Yes	8	0	*	1	0.296*	-
No	18	6				
Non-healthcare or non-medical work experience						
Yes	11	1	*	1	0.370*	-
No	15	5				
Research						
Yes	7	2	*	1	1.00*	-
No	19	4				
The COVID-19 pandemic has affected my experience while in medical school (remote learning, additional calls, etc.)						
Yes (positive or negative impact)	11	3	*	1	1.00*	-
No	15	3				
The COVID-19 pandemic has affected my attitude to pursue a career in hospital medicine and/or critical care medicine						
Yes, (positive impact or negative impact)	9	3	*	1	0.647	-
No impact	17	3				
The COVID-19 pandemic has affected my desire to pursue a career in medicine						
Yes (positive or negative impact)	7	3	*	1	0.346*	-
No	19	3				
Did COVID-19 impact you personally?						
Yes	16	3	*	1	0.666*	-
No	10	3				
Did COVID-19 impact you professionally?						
Yes	16	5	*	1	0.637*	-
No	10	1				
What minimum income level is acceptable to you as a practicing physician?						
Low (under $200,000)	7	3	*	1	0.346*	-
High (equal or above $200,000)	19	3				
Would you like to be employed or be part of a private/group practice?**						
Employed	7	0	*	1	0.245*	-
Private/group practice	8	3				
Do you have a desire to practice in a specific setting?						
Urban	4	2	*	1	1.00*	-
Rural/suburban	9	3				
Unsure**	13	1				
Expected total educational debt (undergraduate+graduate school/medical school)						
Debt low (under $150,000)	16	1	*	1	0.075*	-
Debt high (equal or over $150,000)	10	5				
The pandemic has changed the factors I prioritize when choosing a field of medicine						
Yes	11	3	*	1	1.00*	-
No	15	3				

**Table 4:** The factors (measured on a scale of 0 to 7) associated with choosing Pediatrics (including Pediatrics, Pediatric Oncology, and NICU) as a specialty among medical students at PLFSOM, excluding participants with missing data NICU, Neonatal Intensive Care Unit; PLFSOM, Paul L. Foster School of Medicine

	Mean ranks (those who did not choose)	Mean ranks (those who chose)	Total N	Standardized test statistic	Mann-Whitney U p-value	Effect size
Rate the level of importance this factor weighs in your decision						
It is important for me to choose a field that will provide prestige/respect within the medical community	24.66	28.85	50	0.825	0.422	0.116
It is important for me to choose a field in medicine that will provide a flexible lifestyle	25.43	33.75	53	1.594	0.111	0.218
Please rank your level of concern about the following factors						
Workload/work flexibility	24.95	35.80	53	2.066	0.039*	0.283
Support (personal or professional)	26.01	28.55	52	0.486	0.627	0.067
Control and autonomy	26.00	31.30	53	1.000	0.317	0.137
Maintaining healthy relationships with family, friends, loved ones outside of work	25.67	32.70	53	1.495	0.135	0.205
Documentation and administrative responsibilities	25.52	30.60	52	0.971	0.331	0.134
Liabilities and malpractice	27.72	23.90	53	- 0.717	0.473	0.098

Students who chose IM were more likely to report that COVID-19 did not affect their medical school experience compared with other specialty groups (p=0.015). Among those who chose IM, 18% (3) reported that the pandemic had affected their medical school experience, compared with 63% (12) of those who did not choose IM (Table 5). Other quantitative variables were not associated with choosing IM (Table 6).

**Table 5:** The factors associated with choosing Internal Medicine (including Internal Medicine, Endocrinology, Pulmonology, Internal Medicine and Psychiatry dual, Cardiology, Gastroenterology, and Hematology/Oncology) as a specialty among medical students at PLFSOM, excluding participants with missing data MS, medical students; PLFSOM, Paul L. Foster School of Medicine *Fisher's exact test was done **“Unsure” row was not included in this analysis

Variable	Did not choose Internal Medicine	Chose Internal Medicine	Chi-square	Degrees of freedom	Chi-square p-value	Cramer’s V effect Size
Gender						
Male	6	7	0.2928	1	0.588	0.075
Female	9	7				
Age						
18-26	7	4	1.0071	1	0.316	0.186
27+	8	10				
Ethnicity						
Asian/Asian American+Black/African American	5	5	0.018	1	0.892	0.024
White+Hispanic/Latin American	10	9				
Marital status						
Single	8	8	0.0425	1	0.837	0.038
Married	7	6				
Parental status						
Children	2	2	0.0055	1	0.941	0.014
No children	13	12				
Year of study						
MS1/MS2	6	9	1.710	1	0.190	0.242
MS3/MS4	9	5				
Highest level of education obtained thus far						
Bachelor's degree	11	9	0.277	1	0.599	0.098
Master's degree	4	5				
Do you have prior professional work experience?						
Yes	15	14			Cannot compute	
No	0	0				
How many years of professional work experience?						
<1 year	3	1	*	1	0.597*	-
>1 year	12	13				
How would you categorize your most significant work experience? (choose all that apply)						
Direct patient care						
Yes	12	8	0.614	1	0.433	0.132
No	7	8				
Administrative						
Yes	5	4	0.0079	1	0.929	0.015
No	14	12				
Non-healthcare or non-medical work experience						
Yes	7	5	0.1206	1	0.728	0.059
No	12	11				
Research						
Yes	4	6	1.1513	1	0.283	0.149
No	15	10				
The COVID-19 pandemic has affected my experience while in medical school (remote learning, additional calls, etc.)						
Yes (positive impact or negative impact)	12	3	*	1	0.015*	-
No impact	7	13				
The COVID-19 pandemic has affected my attitude to pursue a career in hospital medicine and/or critical care medicine						
Yes (positive impact or negative impact)	9	5	0.940	1	0.332	0.097
No impact	10	11				
The COVID-19 pandemic has affected my desire to pursue a career in medicine						
Yes (positive impact or negative impact)	5	6	0.504	1	0.477	0.120
No impact	14	10				
Did COVID-19 impact you personally?						
Yes	12	9	0.1727	1	0.678	0.050
No	7	7				
Did COVID-19 impact you professionally?						
Yes	14	10	0.5041	1	0.478	0.070
No	5	6				
What minimum income level is acceptable to you as a practicing physician?						
you as a practicing physician? Low (under $200,000)	7	11	2.2945	1	0.130	0.216
High (equal or above $200,000)	19	12				
Would you like to be employed or be part of a private/group practice?						
Employed	9	2	*	1	0.226*	-
Private/group practice	9	8				
Unsure**	8	13				
Do you have a desire to practice in a specific setting?						
Urban	6	6	0.221	1	0.637	0.065
Rural/suburban	10	7				
Unsure	10	10				
Expected total educational debt (undergraduate+graduate school/medical school)						
Debt low (under $150,000)	14	12	0.0137	1	0.907	0.017
Debt high (equal or over $150,000)	12	11				
The pandemic has changed the factors I prioritize when choosing a field of medicine						
Yes	11	7	0.7402	1	0.390	0.123
No	15	16				

**Table 6:** Factors (measured on a scale of 0 to 7) associated with choosing Internal Medicine (including Internal Medicine, Endocrinology, Pulmonology, Internal Medicine-Psychiatry dual residency, Cardiology, Gastroenterology, and Hematology-Oncology) as a specialty among medical students at PLFSOM, excluding participants with missing data PLFSOM, Paul L. Foster School of Medicine

	Mean ranks (those who did not choose)	Mean ranks (those who chose)	Total N	Standardized test statistic	Mann-Whitney U p-value	Effect size
Please rate the level of importance this factor weighs in your decision						
a. It is important for me to choose a field that will provide prestige/respect within the medical community	21.56	25.81	46	1.089	0.276	0.160
b. It is important for me to choose a field in medicine that will provide a flexible lifestyle	26.79	22.98	49	-0.967	0.334	0.138
Please rank your level of concern about the following factors						
a. Workload/work flexibility	23.31	26.91	49	0.911	0.362	0.130
b. Support (personal or professional)	22.96	26.17	48	0.811	0.418	0.117
c. Control and autonomy	25.81	24.09	49	-0.430	0.667	0.061
d. Maintaining healthy relationships with family, friends, loved ones outside of work	24.98	25.02	49	0.011	0.991	0.001
e. Documentation and administrative responsibilities	21.84	27.39	48	1.404	0.160	0.202
f. Liabilities and malpractice	24.83	25.20	49	0.092	0.927	0.013

Female students were significantly more interested in Surgery than male students (p=0.017), and year one and year two students expressed more interest than year three and year four students (p=0.036). Those interested in Surgery were also more likely to report that the pandemic changed the factors they prioritized when choosing a field of medicine compared with those interested in other fields (p=0.037) (Table 7). Finally, students who preferred a specialty that would provide a flexible lifestyle were less likely to choose Surgery (p<0.001) (Table 8).

**Table 7:** The factors associated with choosing Surgery (including Otorhinolaryngology, Orthopedics, General Surgery, Ophthalmology, and Obstetrics/Gynecology) as a specialty among medical students at PLFSOM, excluding participants with missing data MS, medical students; PLFSOM, Paul L. Foster School of Medicine *Fisher's exact test was done **“Unsure” row was not included in this analysis

Variable	Did not choose Surgery	Chose Surgery	Chi-square	Degrees of freedom	Chi-square p-value	Cramer’s V effect size
Gender						
Male	18	3	*	1	0.017*	-
Female	16	15				
Age						
18-23	4	5	4.228	2	0.121	0.202
24-26	11	8				
27+	19	5				
Ethnicity						
Asian/Asian American & Black/African American	13	4	0.849	1	0.356	0.130
White & Hispanic/Latin American	21	12				
Marital status						
Single	20	12	0.4852	1	0.486	0.099
Married	13	5				
Parental status						
Children	4	0	*	1	0.286*	-
No children	29	17				
Year of study						
MS 1 & MS 2	15	13	4.380	1	0.036	0.295
MS 3 & MS 4	18	4				
Highest level of education obtained thus far						
Bachelor's degree	24	14	*	1	0.510*	-
Master's degree	9	3				
Do you have prior professional work experience?						
Yes	22	13	0.075	1	0.784	0.038
No	12	6				
How many years of professional work experience?						
<1 year	2	3	*	1	0.337*	-
>1 year	20	10				
How would you categorize your most significant work experience? (choose all that apply)						
Direct patient care						
Yes	15	5	2.9473	1	0.086	0.290
No	7	8				
Administrative						
Yes	5	4	0.2767	1	0.599	0.089
No	17	9				
Non-healthcare or non-medical work experience						
Yes	6	6	1.2929	1	0.256	0.192
No	16	7				
Research						
Yes	5	5	0.9913	1	0.319	0.168
No	17	8				
The COVID-19 pandemic has affected my experience while in medical school (remote learning, additional calls, etc.)						
Yes (positive Impact or negative impact)	14	5	1.349	1	0.245	0.161
No impact	19	14				
The COVID-19 pandemic has affected my attitude to pursue a career in hospital medicine and/or critical care medicine						
Yes (positive impact or negative impact)	14	6	0.599	1	0.438	0.107
No impact	19	13				
The COVID-19 pandemic has affected my desire to pursue a career in medicine						
Yes (positive impact or negative impact)	11	8	0.400	1	0.527	0.087
No impact	22	11				
Did COVID-19 impact you personally?						
Yes	19	14	1.3493	1	0.245	0.161
No	14	5				
Did COVID-19 impact you professionally?						
Yes	23	10	1.5144	1	0.218	0.171
No	10	9				
What minimum income level is acceptable to you as a practicing physician?						
Low (under $200,000)	9	9	2.1543	1	0.142	0.210
High (equal or above $200,000)	22	9				
Would you like to be employed or be part of a private/group practice?						
Employed	6	5	*	1	0.199*	-
Private/group practice	14	3				
Unsure**	11	10				
Do you have a desire to practice in a specific setting?						
Urban	11	5	*	1	0.708*	-
Rural & suburban	13	4				
Unsure**	10	10				
Expected total educational debt (undergraduate+graduate school/medical school)						
Debt low (under $150,000)	15	13	2.889	1	0.089	0.233
Debt high (equal or over $150,000)	19	6				
The pandemic has changed the factors I prioritize when choosing a field of medicine						
Yes	8 (44.4%)	10 (55.6%)	4.3365	1	0.037	0.297
No	23 (74.2%)	8 (25.8%)				

**Table 8:** The factors (measured on a scale of 0 to 7) associated with choosing Surgery (including Otorhinolaryngology, Orthopedics, General Surgery, Ophthalmology, and Obstetrics/Gynecology) as a specialty among medical students at PLFSOM, excluding participants with missing data PLFSOM, Paul L. Foster School of Medicine

	Mean ranks (those who did not choose)	Mean ranks (those who chose)	Total N	Standardized test statistic	Mann-Whitney U p-value	Effect size
Please rate the level of importance this factor weighs in your decision						
a. It is important for me to choose a field that will provide prestige/respect within the medical community	24.81	26.63	50	0.436	0.663	0.061
b. It is important for me to choose a field in medicine that will provide a flexible lifestyle	32.13	17.82	53	-3.361	<0.001*	0.461
Please rank your level of concern about the following factors						
a. Workload/work flexibility	29.88	21.84	53	-1.877	0.061	0.257
b. Support (personal or professional)	25.42	28.37	52	0.689	0.491	0.095
c. Control and autonomy	27.69	25.76	53	-0.446	0.656	0.061
d. Maintaining healthy relationships with family, friends, loved ones outside of work	27.59	25.95	53	-0.428	0.669	0.058
e. Documentation and administrative responsibilities	26.33	26.79	52	0.107	0.915	0.014
f. Liabilities and malpractice	25.01	30.55	53	1.274	0.203	0.174

Among students who selected EM, respondents with more than one year of work experience were less likely to choose EM (p=0.020) and were more likely to prefer employment rather than private or group practice (p<0.001) (Table 9). Other quantitative variables were not associated with choosing EM (Table 10).

**Table 9:** The factors associated with choosing Emergency Medicine as a specialty among medical students at PLFSOM, excluding participants with missing data MS, medical students; PLFSOM, Paul L. Foster School of Medicine *Fisher's exact test was done **“Unsure” row was not included in this analysis

Variable	Did not choose Emergency Medicine	Chose Emergency Medicine	Chi-square	Degrees of freedom	p-value	Cramer’s V effect size
Gender						
Male	11	2	*	1	0.662*	-
Female	12	4				
Age						
18-23 & 24-26	8	3	*	1	0.645*	-
27+	15	3				
Ethnicity						
Asian/Asian American & Black/African American	8	2	*	1	1.00	-
White & Hispanic/Latin American	15	4				
Marital status						
Single	13	3	*	1	1.00*	-
Married	10	3				
Parental status						
Children	4	6	*	1	0.059	-
No children	19	6				
Year of study						
MS 1/MS2	13	2	*	1	0.389*	-
MS 3/MS4	10	4				
Highest level of education obtained thus far						
Bachelor's degree	16	4	*	1	1.00*	-
Master's degree	7	2				
Do you have prior professional work experience?						
Yes	23	6			Not computed	
No	-	-				
How many years of professional work experience?						
<1 year	1 (25%)	3 (75%)	*	1	0.020*	-
>1 year	22 (88%)	3 (12%)				
How would you categorize your most significant work experience? (choose all that apply)						
Direct patient care						
Yes	16	4	*	1	1.00*	-
No	12	3				
Administrative						
Yes	7	2	*	1	1.00*	-
No	21	5				
Non-healthcare or non-medical work experience						
Yes	10	2	*	1	1.00*	-
No	18	5				
Research						
Yes	10	0	*	1	0.084*	-
No	18	7				
The COVID-19 pandemic has affected my experience while in medical school (remote learning, additional calls, etc.)						
Yes (positive impact or negative impact)	12	3	*	1	1.00*	-
No impact	16	4				
The COVID-19 pandemic has affected my attitude to pursue a career in hospital medicine and/or critical care medicine						
Yes (positive impact or negative impact)	12	2	*	1	0.676*	-
No impact	16	5				
The COVID-19 pandemic has affected my desire to pursue a career in medicine						
Yes (positive impact or negative impact)	10	1	*	1	0.391*	-
No impact	18	6				
Did COVID-19 impact you personally?						
Yes	15	6	*	1	0.202*	-
No	13	1				
Did COVID-19 impact you professionally?						
Yes	18	6	*	1	0.391*	-
No	10	1				
What minimum income level is acceptable to you as a practicing physician?						
Low (under $200,000)	15	3	*	1	0.723*	-
High (equal or above $200,000)	23	8				
Would you like to be employed or be part of a private/group practice?						
Employed	3	8	*	1	<0.001*	-
Private/group practice	17	0				
Unsure**	18	3				
Do you have a desire to practice in a specific setting?						
Urban	9	3	*	1	0.622	-
Rural/suburban	15	2				
Unsure	14	6				
Expected total educational debt (undergraduate+graduate school/medical school)						
Debt low (under $150,000)	21	5	0.3295	1	0.566	0.082
Debt high (equal or over $150,000)	17	6				
The pandemic has changed the factors I prioritize when choosing a field of medicine						
Yes	14	4	0.0008	1	0.977	0.004
No	24	7				

**Table 10:** The factors (measured on a scale of 0 to 7) associated with choosing Emergency Medicine as a specialty among medical students at PLFSOM, excluding participants with missing data PLFSOM, Paul L. Foster School of Medicine

	Mean ranks (those who did not choose)	Mean ranks (those who chose)	Total N	Standardized test statistic	Mann-Whitney U p-value	Effect size
Please rate the level of importance this factor weighs in your decision						
a. It is important for me to choose a field that will provide prestige/respect within the medical community	22.29	27.36	46	1.114	0.284	0.164
b. It is important for me to choose a field in medicine that will provide a flexible lifestyle	24.51	26.68	49	0.460	0.645	0.065
Please rank your level of concern about the following factors						
a. Workload/work flexibility	26.13	21.09	49	-1.065	0.287	0.152
b. Support (personal or professional)	26.07	19.23	48	-1.452	0.147	0.209
c. Control and autonomy	26.37	20.27	49	-1.273	0.203	0.181
d. Maintaining healthy relationships with family, friends, loved ones outside of work	24.95	25.18	49	0.055	0.956	0.007
e. Documentation and administrative responsibilities	25.07	22.59	48	- 0.527	0.598	0.076
f. Liabilities and malpractice	24.43	26.95	49	0.526	0.599	0.075

None of the variables examined were associated with choosing FM (Table 11 and Table 12).

**Table 11:** The factors associated with choosing Family Medicine as a specialty among medical students at PLFSOM, excluding participants with missing data MS, medical students; PLFSOM, Paul L. Foster School of Medicine *Fisher's exact test was done **“Unsure” row was not included in this analysis)

Variable	Did not choose Family Medicine	Chose Family Medicine	Chi-square	Degrees of freedom	Chi-square p-value	Cramer’s V effect size
Gender						
Male	9	4	*	1	0.144*	-
Female	15	1				
Age						
18-23 & 24-26	9	2	*	1	1.00*	-
27+	15	3				
Ethnicity						
Asian/Asian American+Black/African American	8	2	*	1	1.00*	-
White+Hispanic/Latin American	16	3				
Marital status						
Single	13	3	*	1	1.00*	-
Married	11	2				
Parental status						
Children	4	0	*	1	1.00*	-
No children	20	5				
Year of study						
MS 1/MS2	12	3	*	1	1.00*	-
MS 3/MS4	12	2				
Highest level of education obtained thus far						
Bachelor's degree	18	2	*	1	0.287*	-
Master's degree	6	3				
Do you have prior professional work experience?						
Yes	24	5			Not computed	
No	-	-				
How many years of professional work experience?						
<1 year	4	5	*	1	0.085*	-
>1 year	20	5				
How would you categorize your most significant work experience? (choose all that apply)						
Direct patient care						
Yes	18	2	*	1	0.112*	-
No	10	5				
Administrative						
Yes	6	3	*	1	0.339*	-
No	22	4				
Non-healthcare or non-medical work experience						
Yes	10	2	*	1	1.00*	-
No	18	5				
Research						
Yes	6	4	*	1	0.155*	-
No	22	3				
The COVID-19 pandemic has affected my experience while in medical school (remote learning, additional calls, etc.)						
Yes (positive impact or negative impact)	14	1	*	1	0.198*	-
No impact	14	6				
The COVID-19 pandemic has affected my attitude to pursue a career in hospital medicine and/or critical care medicine						
Yes (positive impact or negative impact)	12	2	*	1	0.676*	-
No impact	16	5				
The COVID-19 pandemic has affected my desire to pursue a career in medicine						
Yes (positive impact or negative impact)	8	3	*	1	0.652*	-
No impact	20	4				
Did COVID-19 impact you personally?						
Yes	15	6	*	1	0.202*	-
No	13	1				
Did COVID-19 impact you professionally?						
Yes	18	6	*	1	0.391*	-
No	10	1				
What minimum income level is acceptable to you as a practicing physician?						
Low (under $200,000)	12	6	*	1	0.140*	-
High (equal or above $200,000)	27	4				
Would you like to be employed or be part of a private/group practice?						
Employed	10	1	*	1	1.00*	-
Private/group practice	14	3				
Unsure**	15	6				
Do you have a desire to practice in a specific setting?						
Urban	11	1	*	1	0.187*	-
Rural/suburban	11	6				
Unsure**	17	3				
Expected total educational debt (undergraduate+graduate school/medical school)						
Debt low (under $150,000)	23	3	*	1	0.157*	-
Debt high (equal or over $150,000)	16	7				
The pandemic has changed the factors I prioritize when choosing a field of medicine						
Yes	14	4	*	1	1.00*	-
No	25	6				

**Table 12:** The factors (measured on a scale of 0 to 7) associated with choosing Family Medicine as a specialty among medical students at PLFSOM, excluding participants with missing data PLFSOM, Paul L. Foster School of Medicine

	Mean ranks (those who did not choose)	Mean ranks (those who chose)	Total N	Standardized test statistic	Mann-Whitney U p-value	Effect size
Please rate the level of importance this factor weighs in your decision						
a. It is important for me to choose a field that will provide prestige/respect within the medical community	16.59	16.00	32	-0.132	0.920	0.023
b. It is important for me to choose a field in medicine that will provide a flexible lifestyle	19.52	11.93	35	-1.818	0.079	0.307
Please rank your level of concern about the following factors						
a. Workload/work flexibility	18.41	16.36	35	-0.489	0.643	0.082
b. Support (personal or professional)	17.48	17.57	34	0.022	1.000	0.003
c. Control and autonomy	18.20	17.21	35	-0.233	0.825	0.039
d. Maintaining healthy relationships with family, friends, loved ones outside of work	18.86	14.57	35	-1.174	0.340	0.198
e. Documentation and administrative responsibilities	17.79	16.17	34	-0.367	0.741	0.062
f. Liabilities and malpractice	18.96	14.14	35	-1.133	0.281	0.191

Finally, for Psychiatry, the only significant finding was that students who were more concerned about personal or professional support were more likely to choose Psychiatry (Table 13 and Table 14).

**Table 13:** The factors associated with choosing Psychiatry as a specialty among medical students at PLFSOM, excluding participants with missing data MS, medical students; PLFSOM, Paul L. Foster School of Medicine *Fisher's exact test was done **“Unsure” row was not included in this analysis)

Variable	Did not choose Psychiatry	Chose Psychiatry	Chi-square	Degrees of freedom	Chi-square p-value	Cramer’s V effect size
Gender						
Male	10	3	*	1	1.00*	-
Female	13	3				
Age						
18-23 & 24-26	10	1	*	1	0.362*	-
27+	13	5				
Ethnicity						
Asian/Asian American+Black/African American	8	2	*	1	1.00*	-
White+Hispanic/Latin American	15	4				
Marital status						
Single	12	4	*	1	0.662*	-
Married	11	2				
Parental status						
Children	4	0	*	1	0.553*	-
No children	19	6				
Year of study						
MS 1/MS 2	14	1	*	1	0.080*	-
MS 3/MS 4	9	5				
Highest level of education obtained thus far						
Bachelor's degree	16	4	*	1	1.00*	-
Master's degree	7	2				
Do you have prior professional work experience?						
Yes	23	6			Not computed	
No	-	-				
How many years of professional work experience?						
<1 year	3	1	*	1	1.00*	-
>1 year	20	5				
How would you categorize your most significant work experience? (choose all that apply)						
Direct patient care						
Yes	16	4	*	1	1.00*	-
No	12	3				
Administrative						
Yes	7	2	*	1	1.00*	-
No	21	5				
Non-healthcare or non-medical work experience						
Yes	10	2	*	1	1.00*	-
No	18	5				
Research						
Yes	9	1	*	1	0.644	-
No	19	6				
The COVID-19 pandemic has affected my experience while in medical school (remote learning, additional calls, etc.)						
Yes (positive impact or negative impact)	15	2	*	1	0.416	-
No impact	15	5				
The COVID-19 pandemic has affected my attitude to pursue a career in hospital medicine and/or critical care medicine						
Yes (positive impact or negative impact)	11	3	*	1	1.00*	-
No impact	17	4				
The COVID-19 pandemic has affected my desire to pursue a career in medicine						
Yes (positive impact or negative impact)	9	2	*	1	1.00*	-
No impact	19	5				
Did COVID-19 impact you personally?						
Yes	16	5	*	1	0.676	-
No	12	2				
Did COVID-19 impact you professionally?						
Yes	19	5	*	1	1.00*	-
No	9	2				
What minimum income level is acceptable to you as a practicing physician?						
Low (under $200,000)	16	2	*	1	0.454	-
High (equal or above $200,000)	24	7				
Would you like to be employed or be part of a private/group practice?						
Employed	10	1	*	1	0.619*	-
Private/group practice	13	4				
Unsure**	17	4				
Do you have a desire to practice in a specific setting?						
Urban	10	2	*	1	0.664	-
Rural/suburban	12	5				
Unsure**	18	2				
Expected total educational debt (undergraduate+graduate school/medical school)						
Debt low (under $150,000)	22	4	*	1	0.716	-
Debt high (equal or over $150,000)	18	5				
The pandemic has changed the factors I prioritize when choosing a field of medicine						
Yes	14	4	*	1	0.707	-
No	26	5				

**Table 14:** The factors (measured on a scale of 0 to 7) associated with choosing Psychiatry as a specialty among medical students at PLFSOM, excluding participants with missing data PLFSOM, Paul L. Foster School of Medicine

	Mean ranks (those who did not choose)	Mean ranks (those who chose)	Total N	Standardized test statistic	Mann-Whitney U p-value	Effect size
Please rate the level of importance this factor weighs in your decision						
a. It is important for me to choose a field that will provide prestige/respect within the medical community	24.01	20.64	46	-0.623	0.549	0.091
b. It is important for me to choose a field in medicine that will provide a flexible lifestyle	24.81	25.83	49	0.201	0.849	0.028
Please rank your level of concern about the following factors.						
a. Workload/work flexibility	23.55	31.44	49	1.548	0.140	0.221
b. Support (personal or professional)	22.32	33.94	48	2.291	0.023	0.330
c. Control and autonomy	23.98	29.56	49	1.082	0.301	0.154
d. Maintaining healthy relationships with family, friends, loved ones outside of work	24.18	28.67	49	0.975	0.408	0.139
e. Documentation and administrative responsibilities	24.54	24.31	48	-0.042	0.968	0.006
f. Liabilities and malpractice	26.14	19.94	49	-1.199	0.245	0.171

## Discussion

Contrary to some other studies [4,5], about 60% of respondents in the present study stated that COVID-19 had no perceived effect on their education. This may be expected because many processes and procedures had returned to normal at TTUHSC El Paso for the study population by the time the survey was administered. In contrast, a prior qualitative study conducted by Krier et al. found that many medical students reported that the COVID-19 pandemic disrupted their learning opportunities. The authors suggested that mentorship and shadowing are important in specialty decision-making and that the loss of these opportunities during the pandemic may have long-lasting effects on medical students’ residency decisions [4]. Similarly, in Krier et al.'s study of medical students in New York City, the majority (59%) believed that COVID-19 influenced their residency or specialty choice. Factors associated with COVID-19 influencing residency choice included having low educational debt (under $99,999), which was associated with higher odds (adjusted odds ratio (OR)=2.23) compared with having no debt, and identifying as another race or ethnicity, which was associated with lower odds (adjusted OR=0.26) compared with identifying as White. In addition, direct personal impact from COVID-19 significantly increased the likelihood that COVID-19 influenced specialty choice (adjusted OR=1.90). Overall, 11.6% of medical students reported changing their top residency choice from before to during or after the pandemic because of concerns about frontline work, work-life balance, and risk of harm [5].

Our study also identified other factors that influence medical students’ residency and career choices. A systematic review by Yang et al. analyzed the interplay of various factors influencing residency choice and found that academic interest played a greater role in developed countries than in developing countries, whereas the importance of prestige was greater in developing countries [6]. In a survey conducted at 15 representative US medical schools, students seeking a high-prestige career were more likely to prefer a non-PC specialty than a PC specialty, and almost half of the students reported that prestige was important when choosing a residency [7]. Similarly, in a cohort study conducted at the University of Connecticut, students interested in surgical specialties were more strongly motivated by higher salary and prestige [8]. In our study, the median importance assigned to choosing a specialty that provides prestige was 4 on a 0-7 scale, indicating moderate importance within our study population. However, neither prestige nor minimum expected annual income was associated with choosing any of the specialties examined.

In our study, student debt was not associated with specialty choice. A study examining the influence of debt on the career decisions of 1991 and 1992 US medical school graduates found complex direct and indirect effects of debt under specific conditions. Educational debt and other financial indicators were associated with choosing PC specialties, such as FM and general IM, and debt from subsidized loan sources was significant among women who chose general IM. However, opposing effects of debt were also observed. For example, students whose debt exceeded a specific threshold were three times more likely to choose FM, whereas for every $10,000 increase in debt above that threshold, the odds of choosing FM decreased by 13% [9].

In our study, the only gender difference in specialty choice was observed for Surgery, with a higher proportion of female students selecting Surgery than male students. However, in Compton et al.'s study of 15 US medical schools, female students were more commonly interested in PC at all three survey time points [7]. Krier et al. argued that, as shadowing and mentorship opportunities became limited during the COVID-19 pandemic, students were forced to choose a specialty with less information than usual. The authors suggested that this may have led more female students toward specialties perceived as more welcoming to women, including PC specialties such as Pediatrics and FM, which may offer greater flexibility for future family responsibilities [4]. Krier et al. also noted that women are often discouraged from pursuing surgical careers because of the lack of female role models in surgery and the perception of poor work-life balance [4]. In contrast, among Canadian medical students, male students were more likely than female students to express interest in surgical specialties, whereas female students were more likely to express interest in FM or another medical specialty [10].

Our study also provided additional insight into the interplay of factors influencing specialty choice. Students interested in Pediatrics were more likely to report greater concern about workload and work flexibility than students interested in other specialties. For these students, prioritizing a healthy workload and work flexibility may outweigh the importance of achieving a higher income to repay educational debt.

In our study, students interested in Surgery were less likely to consider a flexible lifestyle an important factor. Ladha et al. reported that students at the University of Connecticut who were interested in surgical specialties were more strongly motivated by salary and prestige than PC students, who placed greater importance on match confidence and family or location factors. The same study also found that interest in surgical specialties decreased 2.5-fold from year one to year four, whereas interest in PC specialties increased 1.4-fold over the same period [8]. Other studies have shown that the number of US medical students applying for general surgery residencies has declined, possibly because a controllable lifestyle has become an increasingly important factor in specialty selection. Miller et al. suggested that resident work-hour limitations could increase medical students' interest in Surgery [11]. A survey conducted at nine medical schools found that more women were discouraged from choosing Surgery because of the lack of female role models and that students interested in Surgery believed their career choice was influenced by future family considerations [12]. Scott et al. reported that, among Canadian medical students, those interested in surgical or medical specialties were younger, more likely to be single, and more strongly influenced by prestige than students interested in FM. Students interested in Surgery were also less influenced by lifestyle considerations and a broad scope of practice, were less likely to demonstrate a social orientation, and were more likely to be hospital oriented than students interested in FM or another medical specialty [10]. Another Canadian study identified several factors associated with selecting Surgery as the top career choice, including having a relative or friend with a surgical career, volunteering with sports teams, preferring a narrow scope of practice, greater interest in medical care than social issues, an interest in urgent care, and younger age [13]. Consistent with these findings, students in our study who believed that the pandemic changed the factors they prioritized when choosing a specialty and those who were less concerned about a flexible lifestyle were more likely to choose Surgery.

Among our findings, students who were more concerned about personal and professional support were more likely to choose Psychiatry, students who believed that the pandemic affected their medical school experience were less likely to choose IM, and students with more than one year of work experience were less likely to choose EM. A secondary analysis of 2005-2006 Association of American Medical Colleges (AAMC) Graduation Questionnaire data found that, among students choosing EM, lifestyle and residency length were strong influences, whereas mentorship and fellowship opportunities were less influential. The study also suggested that students choosing EM seek a more controllable lifestyle and greater control over their work hours [14].

McDonald et al. found that factors significantly associated with choosing a PC specialty included premedical and medical school research experience, having a family member in PC, student age, and gender. More women than men chose PC, and students who reported that debt influenced their specialty choice were less likely to enter PC. Among men, higher expected salary was negatively associated with entering PC [1]. Osborne et al. found that three factors significantly influenced students' decisions to choose PC: future income, opportunities to work with new technology, and faculty advisors. Higher expected income and opportunities to work with new technology were associated with choosing non-PC specialties, whereas faculty advisors positively influenced students' decisions to choose PC [15]. Another Canadian study found that work-life balance, lifestyle flexibility, the opportunity to promote individual health, the opportunity to establish long-term patient relationships, providing comprehensive care, treating patients and their families, and residency duration significantly influenced the choice of FM or Pediatrics. In contrast, factors associated with choosing a non-frontline specialty included becoming an expert, maintaining a focused scope of practice, having a procedure-focused practice, seeing immediate results, potentially earning a high income, and perceived professional status among colleagues [16]. A narrative review concluded that applicants who self-identified an interest in PC, had a rural background, and were older when entering medical school were more likely to enter PC [17].

A review article reported that having a rural background and planning a career in a disadvantaged or rural area were positively associated with choosing FM, whereas higher parental socioeconomic status was negatively associated with this choice. Students who believed PC was important, had lower income expectations, and did not plan a research career were also more likely to choose FM. Students who attended public schools were more likely to choose FM, and faculty role models served as both positive and negative influences. Students who did not choose FM were more concerned about prestige, lower income, and the broad scope of knowledge required [18]. In Canada, students who identified FM as their first choice tended to be older, more concerned about lifestyle, and more likely to have lived in smaller communities while completing high school. They were also less likely to be hospital-oriented. Furthermore, students who chose FM were much more likely to demonstrate a societal orientation and to prefer a broad scope of practice [19].

Limitations

A limitation of our study is that we assessed students' residency interests at only one time point, whereas studies have shown that students' interests may change over the course of medical school [8]. For example, a study of medical students from 15 US medical schools, in which students completed questionnaires at three time points (during first-year orientation, orientation to clinical rotations, and their senior year), showed that students' interest in surgery decreased over time [7].

Another limitation of this study is that it included only students attending the Paul L. Foster School of Medicine, and the responses were limited to the classes of 2025-2028. These findings are context-specific, and further multicenter validation is needed.

Although all medical students in the classes of 2025-2028 were invited to participate, only 64 students (about 13%) volunteered, and we were unable to increase participation. This limits the generalizability and representativeness of the findings. In addition, the small sample size reduced statistical power and increased the possibility of unstable estimates. Subgroup analyses were also not possible because of the small sample size.

It is also worth noting that, because of multiple comparisons, some results may have reached statistical significance by chance.

By the time this study was conducted, the institution had resumed most of its educational processes in the post-pandemic context. Therefore, response or recall bias may also have affected some of the reported data.

Self-selection bias is also likely because participation was entirely voluntary. In addition, reliance on self-reported intentions rather than actual residency match outcomes is another limitation of this study. As this was a cross-sectional study, no causal inferences can be made.

Implications of the findings

This study may help current medical students develop a framework for identifying the factors that are important to them when considering a medical specialty and encourage them to explore specialties early in their medical education. It also provides medical schools with insights into the factors that are important to students and may help guide mentorship and advising.

Suggestions for future research

As Texas continues to face a shortage of PC physicians, further investigation into the characteristics of medical students who choose PC fields, as well as studies examining interventions that may increase students' interest in pursuing PC specialties, is needed.

Based on the results and limitations of this study, future research should include larger samples from multiple institutions along the US-Mexico border to improve generalizability. Just as the characteristics and needs of patient populations differ across regions, the interests and priorities of medical students when selecting a specialty may also vary according to their region of education or upbringing. Further investigation of these factors could help institutions and communities recruit and retain physicians in underserved areas while promoting job satisfaction and reducing burnout.

Additionally, further research is needed to assess the impact of COVID-19 on specific factors influencing specialty choice. Our study simply asked whether COVID-19 affected participants' decisions but was unable to determine which specific factors were affected or to what extent. It would also be useful to assess the views of students who were most affected by the pandemic during medical school, such as those in the class of 2024 and earlier, along with longitudinal studies evaluating how these factors change throughout residency and beyond.

Theoretical and practical relevance

There are many career-related factors that students must consider when choosing a specialty. However, students are often not formally educated about these issues and may not fully understand their implications, even during residency. Students in this study indicated that they were interested in learning more about topics such as practice settings, employment versus private or group practice, and liability and malpractice considerations. Medical schools have an opportunity to provide interdisciplinary guidance through personalized mentorship or supplementary educational programs to increase students' awareness of these issues. Doing so may provide a more comprehensive understanding of the benefits and implications of a medical career before specialty commitment and may ultimately contribute to greater job satisfaction, higher-quality patient care, and reduced burnout.

## Conclusions

COVID-19 did not appear to affect the factors that medical students near the US-Mexico border considered when choosing a medical specialty. However, among those who reported that the pandemic affected their desire to pursue a career in medicine, the majority described a positive impact. Among the factors assessed, work-life balance appeared to be a high priority for students considering Pediatrics, whereas those who placed less importance on a flexible lifestyle were more likely to choose Surgery. Finally, students who were more concerned about professional and personal support were more likely to choose Psychiatry. Factors such as workload/work flexibility and income may encourage more students to pursue PC specialties; however, given the limitations of this study, these findings should be interpreted with caution.

Future research should include a broader and more geographically diverse population, as well as trainees at more advanced stages of medical education, to better understand the effects of the pandemic, determine whether certain factors are weighed differently across regions of the United States, and evaluate how these priorities change throughout medical school, residency training, and beyond.
